# The fecal carriage rate of extended-spectrum β-lactamase–producing or carbapenem-resistant *Enterobacterales* among Japanese infants in the community at the 4-month health examination in a rural city

**DOI:** 10.3389/fcimb.2023.1168451

**Published:** 2023-06-14

**Authors:** Soichiro Kawata, Shimpei Morimoto, Kosuke Kosai, Yasuhide Kawamoto, Yumiko Nakashima, Yoshitomo Morinaga, Katsunori Yanagihara, Lay-Myint Yoshida, Hiroyuki Moriuchi

**Affiliations:** ^1^ Department of Pediatrics, Nagasaki University Hospital, Nagasaki, Japan; ^2^ Department of Pediatric Infectious Diseases, Institute of Tropical Medicine, Nagasaki University, Nagasaki, Japan; ^3^ Department of Tropical Pediatric Infectious Diseases, Nagasaki University Graduate School of Biomedical Sciences, Nagasaki, Japan; ^4^ Innovation Platform & Office for Precision Medicine, Nagasaki University Graduate School of Biomedical Sciences, Nagasaki, Japan; ^5^ Department of Laboratory Medicine, Nagasaki University Hospital, Nagasaki, Japan; ^6^ Department of Microbiology, Graduate School of Medicine and Pharmaceutical Sciences, University of Toyama, Toyama, Japan; ^7^ Department of Laboratory Medicine, Nagasaki University Graduate School of Biomedical Sciences, Nagasaki, Japan; ^8^ Department of Pediatrics, Nagasaki University Graduate School of Biomedical Sciences, Nagasaki, Japan

**Keywords:** extended-spectrum beta-lactamase-producing *Enterobacterales*, carbapenem-resistant *Enterobacterales*, antimicrobial resistance, antimicrobial stewardship, infants, fecal carriage, *Escherichia coli*, obstetrics facilities

## Abstract

**Background:**

Extended-spectrum β-lactamase–producing *Enterobacterales* (ESBL-E) is a great public health concern globally not only in hospitals but also in the community. To our knowledge, there have been few studies on the prevalence of ESBL-E and much less about carbapenem-resistant *Enterobacterales* (CRE) among children in the community, and there is no such study in Japan despite such situations. This study aimed to clarify their carriage status among Japanese infants in the community by taking the opportunity of the 4-month health checkup.

**Methods:**

This prospective analysis was conducted from April 2020 to March 2021 in Shimabara City, Nagasaki Prefecture, Japan. The research-related items were mailed to all subjects with official documents for the checkup. The fecal samples were obtained from the diaper by guardians beforehand and were collected with the questionnaire and then screened for ESBL-E and CRE by a clinical laboratory company with selective agars followed by identification and confirmation. Only the positive samples were analyzed about resistant genotypes.

**Results:**

One hundred fifty infants aged 4–5 months, over half of the subjects, participated in this study. The overall ESBL-E carriage rate was 19.3% (n = 29), and no CRE carrier was detected among them. All identified ESBL-E were *E. coli* except for one *K. pneumoniae*. A significantly higher carriage rate was recorded among the infants born at “Hospital A” (25.0%) than the others (11.3%). *Enterobacterales* producing CTX-M-9 ± TEM were broadly distributed among the positive samples (65.5%), whereas the CTX-M-1 group was exclusively detected among those from “Hospital A”. Recursive partitioning analysis suggested that delivery facilities might be an important factor for ESBL-E colonization, although the effect could be decreased as they grow. In contrast, no significant effect was observed for other factors such as parent(s) as healthcare worker(s), having a sibling(s), and the mode of delivery.

**Conclusion:**

This study revealed the ESBL-E and CRE carriage status of Japanese infants in the community for the first time, although the setting is somewhat limited. Our findings indicated that environmental factors, especially delivery facilities, influenced ESBL-E colonization among infants aged 4–5 months, implying the need for strengthening countermeasures against antimicrobial resistance at delivery facilities and communities outside the hospitals.

## 1 Introduction

Antimicrobial resistance (AMR) is a serious ongoing problem globally. An estimation from the United Kingdom advocates that AMR in illnesses such as bacterial infections, malaria, tuberculosis, and human immunodeficiency virus will be the first cause of death all around the world in 2050 (O’Neill, 2018). Indeed, patients with urinary tract infections (UTIs) caused by extended-spectrum β-lactamase– producing *Enterobacterales* (ESBL-E) are not rare on the daily clinical scene, even at pediatric clinics in Japan. In addition, we also experienced an outbreak of carbapenemase-producing *Enterobacterales* (CPE) and ESBL-E at the neonatal intensive care units (NICUs) in Nagasaki Prefecture, Japan.

ESBL-E has a higher carriage rate in the community than methicillin-resistant *Staphylococcus aureus.* The global carriage rate of ESBL-E in the community was reported as 16.5% during the period between 1 January 2000 and 13 February 2020. It is increasing continuously from 2.6% (95% CI, 1.6–4.0) in 2003–2005 to 21.1% (95% CI, 15.8%–27.0%) in 2015–2018 and estimated as a 1.5% yearly increase by linear regression analysis (Bezabih et al., 2021). Among six WHO regions (i.e., Southeast Asia, Western Pacific, Africa, Eastern Mediterranean, Americas, and Europe), the Southeast Asian Region had the highest prevalence at 27% (95% CI, 2.9%–51.3%) based on four studies from India, Thailand, and Nepal, whereas the European Region had the lowest at 6.0% (95% CI, 4.6%–7.5%) based on 19 studies.

The ESBL-E carriage rate in Japan was reported to be 6.4% among 218 stool specimens obtained from adult volunteers (Luvsansharav et al., 2011) and 7.5% in 67 stool samples acquired from medical students in 2011 (Niki et al., 2011). There have been subsequent reports of ESBL-E carriage rates among adults in Japan, including 4.8% of 4,314 stool specimens obtained from 2,563 food handlers (Nakane et al., 2016), 12.5% of 257 hospitalized patients and 8.5% of 496 workers at 25 meal supply centers in the same community without significant difference (Nakamura et al., 2016), 12.2% of 263 persons who underwent routine medical checkups (Higa et al., 2019), and 9.7% of 547 healthy individuals (Masui et al., 2022), whereas there have been only a few studies on ESBL-E carriage rates among Japanese children, including 12% of 50 inpatients at a pediatric tertiary care hospital (Minami et al., 2012) and 12.5% of 256 pediatric patients with gastroenteritis among 11 hospitals in Mie Prefecture (Nagai et al., 2017). The worldwide prevalence of ESBL-E carriage in children has been reportedly variable, such as 74% of neonates and 59% of children in Ethiopia’s largest tertiary hospital (Desta et al., 2016), 50.4% of hospitalized patients and 11.6% of children in the community in Tanzania (Tellevik et al., 2016), 2.3% of asymptomatic nursery children in Germany (Harries, et al. 2016), and 3.5% of healthy children in the United States (Islam et al., 2018). The ESBL-E carriage rates have shown an increasing trend even among children in the community. The French study showed the rate to be 7.6% of 1886 children aged 6–24 months with a tendency to increase from 2.2% in 2010 to 10.8% in 2016 (Birgy et al., 2016). The study from Sweden reported that 16.8% of 334 children aged 13–45 months were positive in 2016, over a six-fold increase from 2.6% in 2010 (Kaarme et al., 2018).

Meanwhile, relatively few studies have examined the community-acquired carriage of carbapenem-resistant *Enterobacterales* (CRE). In a review article analyzing 15 studies, the rates of the community-associated or community-onset CRE globally were as various as 0.04%–29% in 10 studies across the world, and five studies did not detect CRE (Kelly et al., 2017). These rates should not be interpreted immediately as the community-acquired carriage rates because most of those reports were not based on the research in the community but on the studies conducted at clinics or estimated from the rates of hospitalized patients on admission. In any case, the results suggest that the CRE threat is not limited to the hospital but is spreading to the community as well. For example, the aforementioned report studying hospitalized patients of all ages in Ethiopia revealed five CRE carriers, all of whom were children. Two of them carried *Klebsiella pneumoniae* carbapenemase –producing CPE (Desta et al., 2016).

To our knowledge, there are no data about the ESBL-E carriage rate of children in the community in Japan and about the CRE carriage rate of non-hospitalized individuals whether adults or children. We conducted this study to reveal these carriage rates among infants in the community in a rural area and to determine associated factors in this area by analyzing the questionnaires.

## 2 Materials and methods

### 2.1 Study setting

This prospective study was performed between April 2020 and March 2021 in Shimabara City, Nagasaki Prefecture, the westernmost part of Japan (**Figure S1**). This city has an estimated population of 43,556 people with live births of 280 neonates in January 2020 and 43,228 people with 295 neonates in January 2021, according to information from Nagasaki Prefecture (https://www.pref.nagasaki.jp/shared/uploads/2021/05/1622446419.pdf; https://www.pref.nagasaki.jp/shared/uploads/2022/05/1653363988.pdf).

ESBL-E or CRE carriage was defined with the positive results of both screening cultures and identification of the resistant genotypes using the methods described below.

This study was approved by the Human Ethics Committee of Nagasaki University Graduate School of Biomedical Sciences.

### 2.2 Study subject

To reveal the actual carriage rate of ESBL-E and CRE among healthy children, we took advantage of the opportunity for a 4-month health checkup, which all children were required to have in Japan. All subjects for the checkup received the supporting documentation, the informed consent documents, questionnaires, and kits for stool sample collection with other documents related to the health checkup through the Shimabara Healthcare Center. All infants with the approval of their parents or guardians were included in this study except those who could not take stool samples or those who were regarded as inappropriate for other reasons.

### 2.3 Sample processing and data collection

All samples were obtained from the stool on the diaper with two separate swabs including Cary-Blair media for culture named Seed swab γI (Eiken Chemical, Ltd.) at each home mainly by parents following the instruction text or the video created originally and uploaded on YouTube^®^ presented on the supporting documentation beforehand of visiting the health checkup (**Figure S2**). These stool samples were collected at the Shimabara Healthcare Center on the day of the checkup. If the parents of the participant could not bring the sample but wanted to join the study, then we asked them to take it to the reception for the Pediatric Department at the Shimabara Prefectural Hospital. Every sample was retrieved and screened for ESBL-E and CRE with 48-h aerobic incubation under 35°C in CHROMagar™ ESBL and CHROMagar™ mSuperCARBA (Kanto Chemical Co., Inc.), respectively, at a major Japanese clinical laboratory company. The former yielded colonies of the following colors: mauve red, metallic blue with or without a red halo, and brown with a halo, and the latter yielded those of the following colors: mauve red and metallic blue. Hence, we picked up one colony each from different color groups from these selection agars. Incubation with 5% Trypto-casein Soy Agar was also performed in parallel for each culture to ensure non-contamination and to use for retesting or additional examination. After the bacterial identification and drug susceptibility test using MALDI-TOF MS (matrix-assisted laser desorption/ionization–time-of-flight mass spectrometer) of MALDI Biotyper^®^ microflex^®^ (Bruker) and Dry Plate Eiken (Eiken Chemical, Ltd.) based on the criteria of CLSI M100S-22, respectively, the ESBL production and CRE characteristic were confirmed with the following methods for all screening-positive samples. ESBL-E was identified when Cica Beta Test I was positive and CVA was negative. If Cica Beta Test was inconclusive, then clavulanic acid added double disc synergy test (DDST) test was performed with cefotaxime and cefpodoxime. CRE was identified under the criteria stipulated by the Japanese Infectious Disease Control Law in which the minimum inhibitory concentration (MIC) for meropenem is 2 µg/ml or higher or the MIC for imipenem is 2 µg/ml or higher, and the MIC for cefmetazole is 64 µg/ml or higher.

Only the positive samples were returned and stored in the Microbank™ (Pro Lab Diagnostics, Inc.) at the −70°C deep freezer placed at the Department of Pediatrics, Nagasaki University Hospital.

The following data were obtained from the questionnaires: gender, birth date, sampling date, gestational age, birth weight, mode of delivery, delivery facility, feeding methods within the first week of birth and around 4 months old, hospitalized history (cause of admission, hospital, antibiotic treatment, and length of hospital stay), siblings, ESBL-E or CRE outbreak exposure of siblings, parental occupation (healthcare worker or else), parental history of staying abroad, and parentally history of hospitalization abroad. The birth date was used for calculating age in days at the time of the health checkup.

### 2.4 Identification of the resistant genomic pattern

The genotypes of ESBL were detected with TaKaRa PCR Thermal Cycler Dice^®^ Gradient (TaKaRa Bio, Inc.) by using Cica Geneus^®^ ESBL Genotype Detection KIT2 (Kanto Chemical Co., Inc.) following its instruction manual. DNA extraction was demonstrated with the boiling method using Chelex (Bio-Rad Laboratories, Hercules, CA, USA) following the previous report with minor modifications (Motoshima et al., 2010). The targeted genes are *bla*_CTX-M (-1, -2, -8, -9, and -25 group)_, *bla*_CTX-M chimera_, *bla*_GES (ESBL type)_, *bla*_TEM_, and *bla*_SHV_. Each abbreviation indicates the following: CTX-M, cefotaximase-Munich; GES, Guiana extended-spectrum β-lactamase; TEM, temoneira; and SHV, sulfhydryl variable.

### 2.5 Data analysis

All the data obtained from the questionnaires were entered into a Microsoft^®^ Excel^®^ (Microsoft Corporation) worksheet at the venue of the health checkup immediately after the collection, and the parent was asked to fill in if anything remained blank or appeared obscure. JMP^®^ Pro 16.0.0 (SAS Institute, Inc.) was used for every statistical analysis. Fisher’s exact test was used to analyze categorical data, and a t-test was used for continuous data. The two-sided *p*-values <0.05 were considered statistically significant. Hence, the objectives of the presenting study were exploratory ones, the statistical significance cannot be interpreted that it is valid to reject the corresponding null hypothesis.

In analyses of the association between two binomial variables, the *p*-values were obtained via Fisher’s exact test and presented to describe the extent of statistical significance of the association. In the analysis, the candidate of the predictor was a continuous variable; instead of Fisher’s exact test, the t-test was used to obtain *p*-values. To determine associated factors among the background of the study subjects among infants in the community, the odds ratios for ESBL-E and CRE carriages and the confidence intervals were calculated. The 95% confidence intervals of binomial probabilities were obtained using the Wilson score method.

In addition to those, recursive partitioning analysis was conducted to describe the bias of background factors in carriers and non-carriers. The nodes in the tree were generated using the likelihood ratio chi-square. The splitting was stopped with the following criteria: No branching increases the likelihood ratio chi-square in the descendent nodes, the number of individuals allocated into nodes containing positive subjects is less than 10, or it is difficult to interpret the results and make hypotheses.

## 3 Results

### 3.1 Number of participants

The research documents and sampling kits were sent to 271 subjects, and 150 infants (55.4%) were eligible to participate in this study except for one infant whose parent had difficulty in informed consent and answering the questionnaire.

### 3.2 Characteristics of the participants

The characteristics of the participants are summarized in **Table 1**. The percentages of preterm (3.3%) and low birth weight infants (5.3%) were lower than those previously reported from Japan (5.7% and 9.4%, respectively) (Isayama, 2019; Mine et al., 2021). Some of the characteristics included too few subjects to assess the impact on ESBL-E carriage, such as “antibiotic treatment during hospitalization”, “sibling’s stay in a relevant ward during the outbreak”, and “parental stay abroad”.

**Table 1:** Characteristics of the participants.

Characteristic		No.	%
Sex	Male	72	48.0
	Female	78	52.0
Age	Median (IQR), day	146 (134 to 160)	
The period from sampling to collection	Median (IQR), day	−1 (−1 to 0)	
Gestational age	Median (IQR), week	39 (38 to 40)	
Birth weight (BW)	Median (IQR), g	3,101 (2,803.5 to 3,376.0)	
Preterm infants		5	3.3
Low birth weight infants (BW below 2,500 g)		8	5.3
Delivered by cesarean section		35	23.3
Completely bottle-fed, perinatally		2	1.3
Completely bottle-fed, currently		33	22.0
History of hospitalization		18	12.0
	Antibiotic treatment	6	4.0
	Unknown	4	2.7
Sibling(s)	Yes	103	68.7
	The sibling’s hospital stay during the outbreak*1	1	1.0
Mother is a healthcare worker	Currently	31	20.7
	In 3 years	8	5.0
	No	111	74.0
Father is a healthcare worker	Currently	17	11.3
	In 3 years	1	0.7
	No	128	85.3
	Not living with father	4	2.7
Mother or father is a healthcare worker	Currently or in 3 years	42	28.0
Parental stay abroad*2	Yes	1	0.7
Parental history of hospitalization abroad	Yes	0	0

*1 The sibling stayed in the perinatal ward during the ESBL-E outbreak in the NICU of a particular facility.

*2 refers more than 2 weeks.

### 3.3 Carriage situation of ESBL-E and CRE

Each participant provided two samples for screening ESBL-E and CRE, and all 300 specimens except for those two from the excluded participant were eligible for testing. ESBL-E was detected from a total of 29 samples (19.3%), whereas no CRE-positive samples were found. Among the ESBL-E positive samples, 28 (96.6%) were *Escherichia coli* and one was *Klebsiella pneumoniae*. One *K. oxytoca* was identified through the screening but excluded because any relevant genotype was detected.

**Figure 1:**
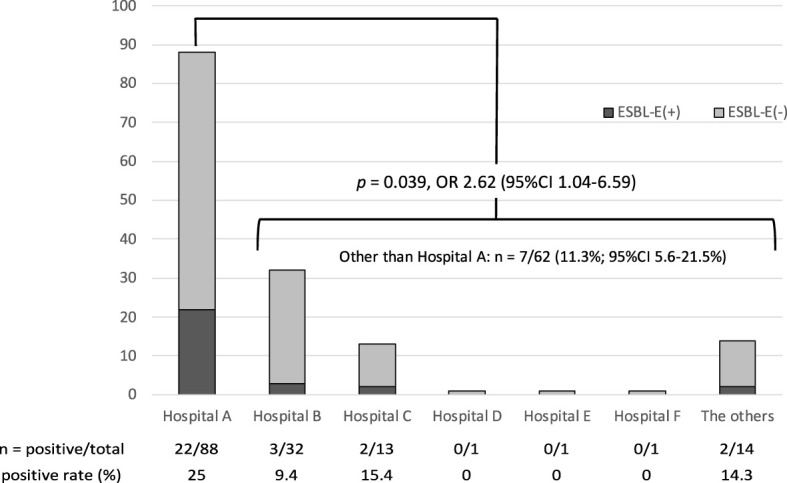
The number of ESBL-E–positive and ESBL-E–negative samples by the birth hospital.

**Table 2** shows the carriage rates according to the background factors, and **Figure 1** exhibits the carriage rates according to the delivery facilities. There was no visible difference in the backgrounds between carriers and non-carriers except for their delivery facilities. “Hospital A”, accounting for the largest number of deliveries in the study site, had more than double and significantly higher ESBL-E prevalence than that of the other facilities (22/88 vs. 7/62, 25.0%; 95% CI, 17.1–35.0% vs. 11.3%; 95% CI, 5.6–21.5%; *p* = 0.039; OR, 2.62; 95% CI, 1.04–6.59).

**Table 2:** ESBL-E carriage rates according to the background factors.

Variables		ESBL-E positive n = 29		ESBL-E negative n = 121	
		No.	%	No.	%
Sex	Male	14	48.3	58	47.9
	Female	15	51.7	63	52.1
Age	Median (IQR), days	137 (130.5 to 165.0)		146 (134.5 to 160.0)	
The period from sampling to collection	Median (IQR), days	0 (−1 to 0)		−1 (−1 to 0)	
Gestational age	Median (IQR), weeks	39 (38.5 to 40.0)		39 (38.0 to 40.0)	
Birth weight (BW)	Median (IQR), grams	3,130 (2,821.0 to 3,413.0)		3,078 (2,800.0 to 3,321.0)	
Preterm infants		1	3.5	4	3.3
Low birth weight infants (BW below 2,500 g)		2	6.9	6	5.0
Delivered by cesarean section		6	20.7	29	24.0
Completely bottle-fed, perinatally		0	0.0	2	1.7
Completely bottle-fed, currently		7	24.1	26	21.5
History of hospitalization		3	10.3	15	12.4
	Antibiotic treatment	0	0.0	6	40.0
	Unknown	0	0.0	4	26.7
Sibling(s)	Yes	22	75.9	81	66.9
	The sibling’s hospital stay during the outbreak*1	1	1.0	0	0.0
Mother is a healthcare worker	Currently	5	17.2	26	21.5
	In 3 years	2	6.9	6	5.0
	No	22	75.9	89	73.6
Father is a healthcare worker	Currently	3	10.3	14	11.6
	In 3 years	0	0.0	1	0.8
	No	26	89.7	102	84.3
	Not living with father	0	0.0	4	3.3
Mother or father is a healthcare worker	Currently or in 3 years	7	24.1	35	28.9
Parental stay abroad*2	Yes	0	0.0	1	0.8

*1 The sibling stayed in the perinatal ward during the ESBL-E outbreak in the NICU of a particular facility.

*2 refers more than 2 weeks.

### 3.4 Recursive partitioning analysis

**Figure 2:**
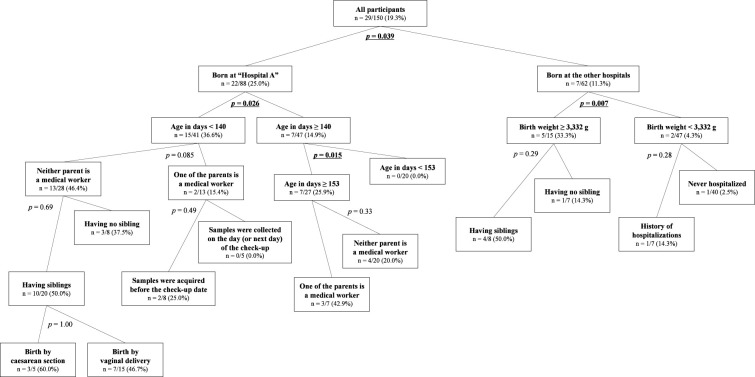
The tree of recursive partitioning analysis about ESBL-E carriage among the participants. The number in each node means the prevalence of ESBL-E carriage [i.e., n = positive/total (carriage rate)]. The *p*-values shown beside each branch were presented not for rejection of the null hypothesis but for exploratory purposes, and those that are lower than 0.05 are emphasized with bold and are underlined.

Recursive partitioning analysis was performed to describe background factor bias in ESBL-E carriers and non-carriers as presented in **Figure 2**. All factors obtained from the questionnaire except for the sub-answers (i.e., antibiotic usage and the duration of hospital stay) were included in the explanatory variables. To better reflect the actual situation, “delivery facilities” was arranged for “Hospital A” and others, and “occupation” was changed to whether one of the parents is currently or has been in the medical profession within the past 3 years or not and was included in the explanatory variables. The first branch was the “delivery facilities” (“Hospital A” and “The other facilities”) with a significant difference between the nodes as the most major contributors to the tree split (statistical values are the same as described above in 3.3). The descendent nodes created under the node “those from ‘Hospital A’” was the “days of age” with a cutoff of 140 days (15/41 vs. 7/47, 36.6% vs. 14.9%, *p* = 0.026, OR 3.30; 95% CI, 1.21–8.96). The descendent nodes created under the “those from the other facilities” was “birth weight” with a cutoff of 3,332 g (5/15 vs. 2/47, 33.3% vs. 4.3%; *p* = 0.007; OR, 11.25; 95% CI, 2.14–57.53). The nodes of “days of age” with a cutoff of 153 days among those older than 140 days from “Hospital A” (7/27 vs. 0/20, 25.9% vs. 0.0%; *p* = 0.015; OR, ∞; 95% CI, 1.68 –∞) were the only descendent node of above nodes with p < 0.05.

### 3.5 Analysis of resistant genes

The genotypes of ESBL were detected for all 30 positive samples except for one of *K. oxytoca* (**Table 3**). The CTX-M-9 group was the most predominant genotype accounting for 16 *E. coli* isolates, followed by the CTX-M-1 group with the TEM gene (n = 7), CTX-M-9 group with TEM gene (n = 3), CTX-M-1 group (n = 1), and TEM gene (n = 1). The only *K. pneumoniae* isolate harbored SHV with the TEM gene.

**Table 3:** ESBL genotypes identified among the screening-positive samples.

ESBL genotypes	Samples (n)	Rate (%)
CTX-M-1 group	1	3.33
CTX-M-1 group, TEM	7	23.33
CTX-M-9 group	16	53.33
CTX-M-9 group, TEM	3	10.00
Not Detected *1	1	3.33
SHV, TEM *2	1	3.33
TEM	1	3.33
Total	30	100.0

*1 refers to one *K. oxytoca* isolate.

*2 refers to one *K. pneumoniae* strain.

In addition, as shown in **Table 4** and **Figure S3**, a total of eight CTX-M-1 group *E. coli* were dominantly detected in the participants born at “Hospital A” with statistical significance compared with those from the other facilities (8/88 vs. 0/62, 9.1% vs. 0.0%; *p* = 0.021; OR, ∞; 95% CI, 1.58–∞) and of 11 TEM *E. coli* as well (10/88 vs. 1/62, 11.4% vs. 1.6%; *p* = 0.027; OR, 7.82; 95% CI, 1.24–48.37), whereas CTX-M-9 group isolates, which accounted for 19 (65.5%) of the 29 ESBL positives, were widely distributed regardless of the delivery facilities (12/88 vs. 7/62, 13.6% vs. 11.3%; *p* = 0.81; OR, 1.24; 95% CI, 0.47–3.26).

**Table 4:** The differences in detected ESBL genotypes about *E. coli* between birth facilities.

ESBL genotypes	Hospital A n (%*)	The others n (%*)	Statistical values
CTX-M-1 group	8 (9.1)	0 (0.0)	*p* = 0.021, OR ∞ (95% CI 1.58–∞)
CTX-M-9 group	12 (13.6)	7 (11.3)	*p* = 0.81, OR 1.24 (95% CI 0.47–3.26)
TEM	10 (11.4)	1 (1.6)	*p* = 0.027, OR 7.82 (95% CI 1.24–48.37)
Overall ESBL positive *E. coli*	21 (23.9)	7 (11.3)	*p* = 0.058, OR 2.46 (95% CI 0.99–6.07)

* These rates indicate the prevalence of the arbitrary genotype of ESBL-positive *E. coli* among all participants born in each hospital.

## 4 Discussion

To the best of our knowledge, this is the first study about the ESBL-E carriage situation among infants in the community of Japan, as well as about the CRE carriage for non-hospitalized people in Japan although no CRE carrier was detected. In the present study, the ESBL-E carriage rate of infants aged 4 to 5 months in Shimabara city was 19.3%. The infants born at “Hospital A” had a significantly high carriage rate of 25.0% compared with 11.3% in those born at other delivery facilities. The overall prevalence was >1.5-fold higher than those from the previous studies among healthy adults or pediatric samples from hospitals in Japan (Luvsansharav et al., 2011; Niki et al., 2011; Minami et al., 2012; Nakamura et al., 2016; Nakane et al., 2016; Nagai et al., 2017; Higa et al., 2019; Masui et al., 2022); however, it is worthy to note that the ESBL-E prevalence at delivery facilities other than “Hospital A” was comparable to those from the previous studies.

The fecal carriage of ESBL-E can cause UTIs. The annual proportion of ESBL-E among pediatric UTIs in Japan has been reported to be 3.6%–21.1% (Nagata et al., 2015; Yanai et al., 2019; Sakata et al., 2021; Okumiya et al., 2022), except for a study from Shimane Prefecture reporting over 50% prevalence (Horie et al., 2019). These data should not be immediately interpreted as the ESBL-E carriage rate in the community because the specimens obtained at hospitals tend to show higher carriage rates (Bezabih et al., 2022). However, clinicians should be aware of the ESBL-E prevalence among patients with UTIs at their hospitals for appropriate treatment strategies. For example, in high-prevalence hospitals, intravenous administration of cefmetazole or flomoxef can be selected as the initial therapy for febrile UTI at hospitals with high ESBL-E prevalence in Japan (Matsumura et al., 2015; Fukuchi et al., 2016). Moreover, the escalation to meropenem without waiting for culture results may not be overtreatment, depending on the clinical course of UTI and the prevalence of ESBL-E because 5%–10% of UTIs with gram-negative rods are complicated by bacteremia (Schnadower et al., 2010; Sakata et al., 2021).

Several studies have shown that the younger the child, the higher the carriage rate (Tellevik et al., 2016; Cheng et al., 2022), and the results of our present study suggest that children aged around 4 months may be susceptible to environmental factors, especially the delivery facilities in which they were born. The recursive partitioning analysis indicated that the birth facility significantly contributed to ESBL-E carriage among our study population. Because previous studies have not included “delivery facility” in the survey items, our finding needs to be examined by future studies. Interestingly, further splitting suggested a slightly decreasing trend in the carriage rate over time, and other factors such as the occupation of their parents as medical workers, having siblings, or the mode of delivery did not show statistical significance for the ESBL-E carriage status, although these might be the results of the limitations about our study subjects. We are planning to conduct a similar study on the ESBL-E carriage for the same population when they became age 3 shortly, which will hopefully reveal changes in the carriage status.

In the present study, the CTX-M-9 group was broadly detected among infants born at various delivery facilities, whereas the CTX-M-1 group was exclusively detected among those born at “Hospital A” with statistical significance, strongly suggesting environmental differences among birth facilities and the possibility of CTX-M-9 spreading in the community outside hospitals. In Japan, these two genotypes are common even in non-clinical specimens (Masui et al., 2022; Sekizuka et al., 2022). In particular, the CTX-M-9 group became dominant over the CTX-M-2 group, which was the most prevalent in the early 2000s (Suzuki et al., 2009). These results emphasize the need for action against AMR not only in hospitals but also in the community and birth facilities.

There are several limitations to our study. First, unlike other studies conducted in medical institutions, the samples were collected by the parent, who might technically be less reliable. To address this issue, we have not only provided an instruction manual simply explaining precautions for specimen collection but also directly confirmed that a sufficient amount of stool adhered to the swabs at the time of collection before handing them over to a clinical laboratory company. Contamination from the environment, such as the surface of diapers, is not expected to significantly affect test results, as the number of bacteria in the stool should be overwhelmingly larger.

Second, parents have not been tested for their carriage status for two reasons: first, performing it would make them hesitate to participate, and, second, submission of plural specimens would increase the risk of sample mix-up.

Third, the results obtained in our study are only from infants aged around 4 to 5 months in a rural city in Japan; therefore, the external validity of the results remains to be assessed. In addition, it is impractical to model the mechanisms of harboring ESBL-E and the network of background factors, thus, we performed the recursive partitioning analysis but not multivariable regression analyses. Future studies in Japan are awaited including the similar survey that we are planning to perform at the age of three as mentioned above.

Finally, because of the influence of the COVID-19 pandemic, the health checkup in May 2020 was canceled and allocated to another month’s checkup schedule. Hence, the ages in months of some participants were slightly older than 4 months. However, the data still reflect the prevalence of carriage during early infancy in Shimabara City and showed a slightly decreasing trend in the carriage rate over time as a by-product.

In conclusion, our study revealed the ESBL-E carriage rate as 19.3% and no CRE carriage among infants aged 4 to 5 months in a rural city in Japan. This is the first report on the ESBL-E carriage rates in children in the Japanese community and the CRE carriage rate in any non-hospitalized individuals in Japan. The ESBL-E carriage rate appeared to be associated with delivery facilities: The infants born at “Hospital A” had a higher carriage rate (25.0%) than those born at other facilities (11.3%) and CTX-M-1 was detected only in those from “Hospital A” among the positive samples. Our results suggested that infants aged 4 to 5 months may be more prone to acquire ESBL-E due to environmental factors, especially their delivery facilities. These findings highlighted the importance of AMR countermeasures not only in hospitals but also in the community and maternity facilities.

A part of this study was presented at the 10th Asian Congress of Pediatric Infectious Diseases (Seoul, October 2022).

## Data availability statement

The raw data supporting the conclusions of this article will be made available by the authors, without undue reservation.

## Ethics statement

The studies involving human participants were reviewed and approved by the Human Ethics Committee of Nagasaki University Graduate School of Biomedical Sciences. Written informed consent to participate in this study was provided by the participants’ legal guardian/next of kin.

## Author contributions

Study design: SK, SM, YN, YM, KY, and HM. Data collection: SK. Data interpretation: SK, SM, KK, and HM. Genotype identification: YK and KK. Statistical analysis: SK and SM. All authors contributed to the initial draft of the manuscript. All authors contributed to the article and approved the submitted version.
