# Supplementary figures and images for: The fecal carriage rate of extended-spectrum β-lactamase–producing or carbapenem-resistant Enterobacterales among Japanese infants in the community at the 4-month health examination in a rural city
Source: Front Cell Infect Microbiol. 2023 Jun 14;13:1168451. doi: 10.3389/fcimb.2023.1168451 (PMC10305779; doi:10.3389/fcimb.2023.1168451)

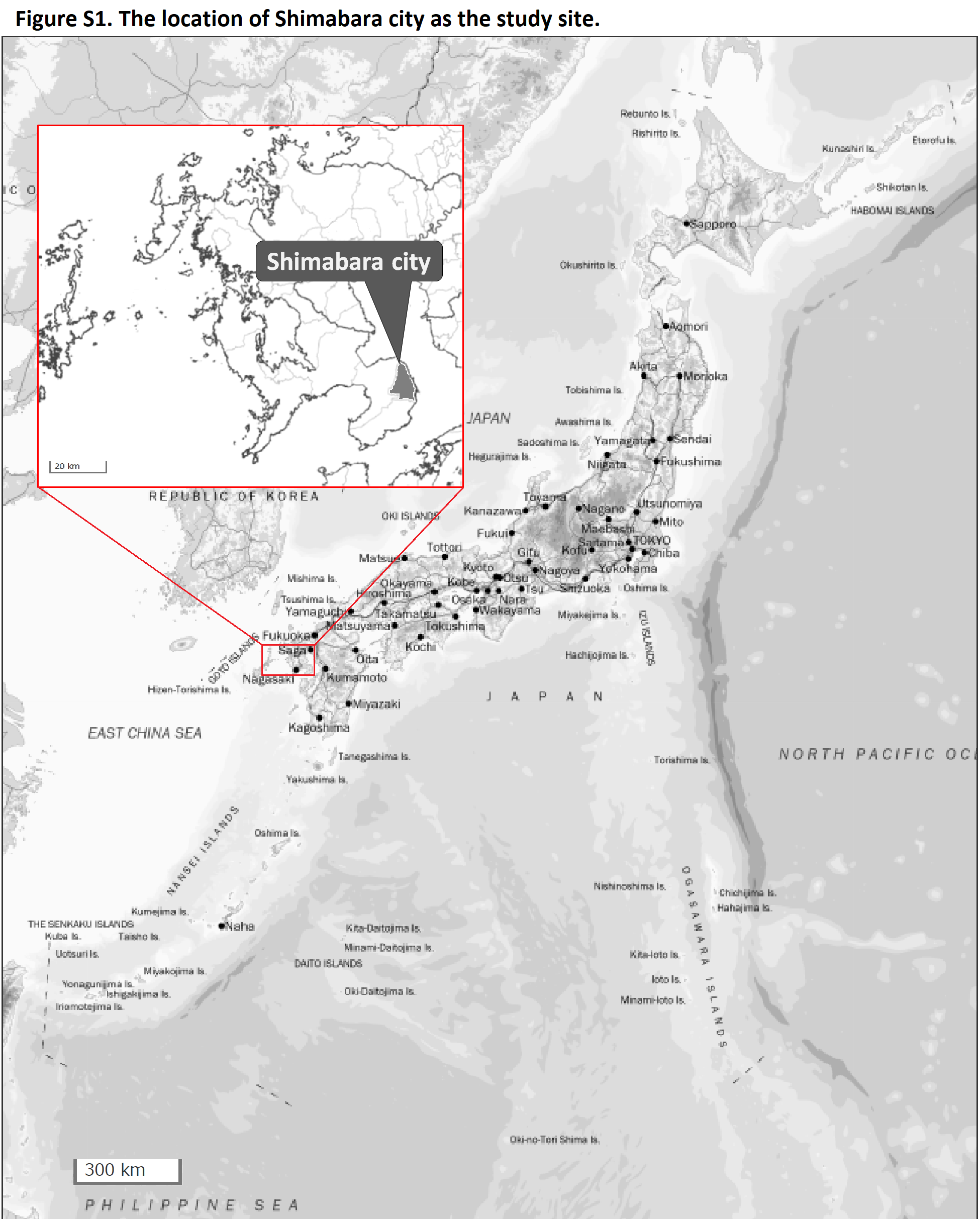

Supplement: Supplementary file 1 [file Image_1.tif]
