# Supplementary material for: The fecal carriage rate of extended-spectrum β-lactamase–producing or carbapenem-resistant Enterobacterales among Japanese infants in the community at the 4-month health examination in a rural city
Source: Front Cell Infect Microbiol. 2023 Jun 14;13:1168451. doi: 10.3389/fcimb.2023.1168451 (PMC10305779; doi:10.3389/fcimb.2023.1168451)
Supplement: Supplementary file 2 [file DataSheet_1.pdf]

Figure S2. The instruction for the participants about collecting the samples. (translated into English)

For those who agreed to participate in this research.

This instruction is also available on YouTube!
https://youtu.be/r0R66HxhXVc

How to take the samples

Please touch only the handle of the swab.<br>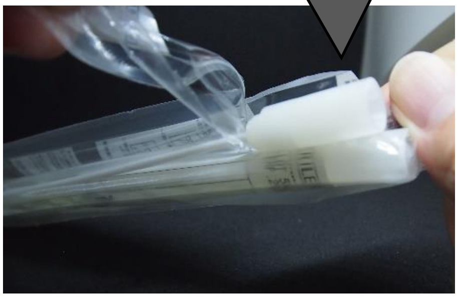	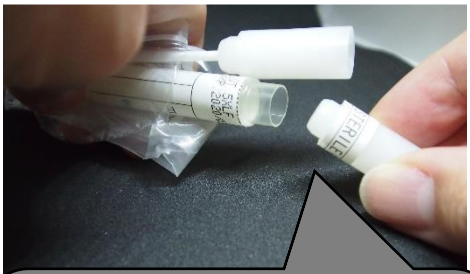<br>Please do not forget to open the lid in advance.<br>__*Especially for the 2nd one__	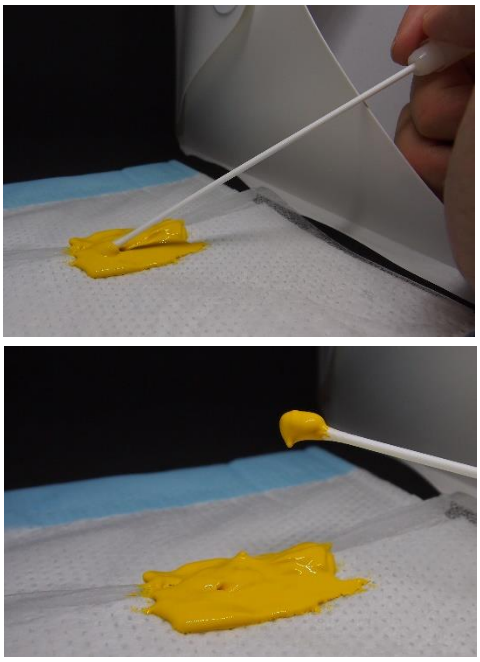
1. Remove the package.	2. Open the lid of the container.	3. Firmly apply the stool to the swab.
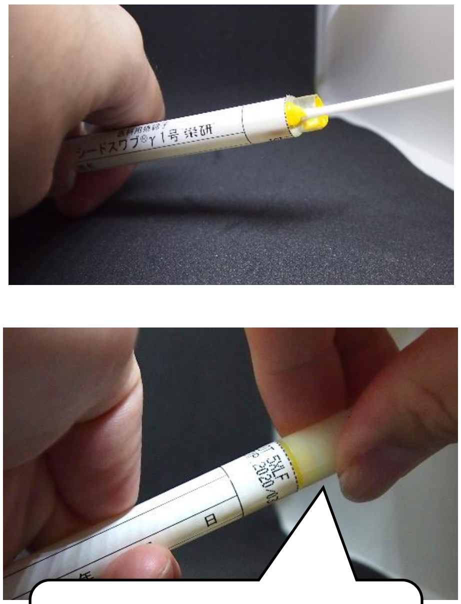<br>Please confirm the swab is inserted steadily.	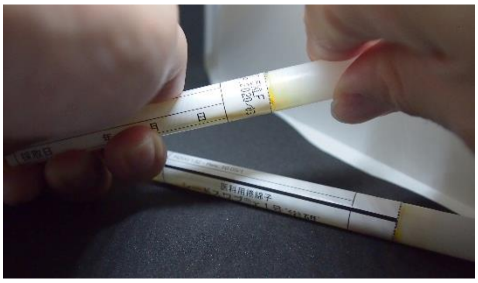	Please peel off the two long papers on the reverse.<br>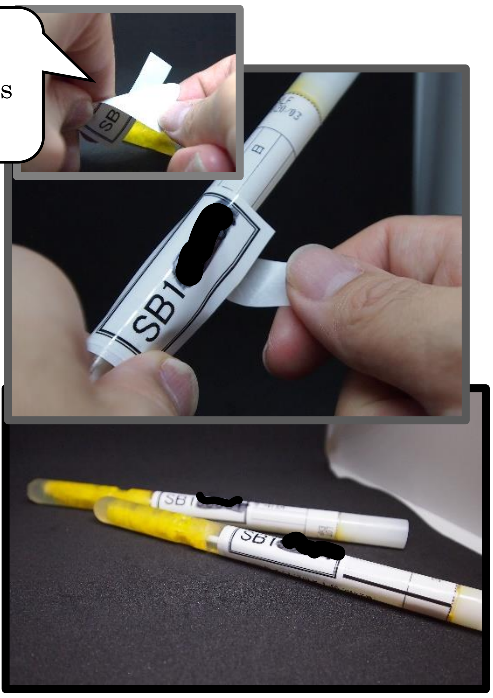
4. Insert it into the container.	5. Process the second one according to 1 to 4.	6. Finish by applying the numbering stickers.

<u>Please answer the questionnaire</u> and <u>bring ALL of the below</u> to the venue (Shimabara Healthcare Center) on the day of the checkup.
I. two collected samples II. questionnaire III. consent form

6 numbering stickers should be used for the following: two for the samples, one for the questionnaire, and another one for the consent form. The rest of the two are for spare and your copy which is required to keep.
